# Exploring Metadata Catalogs in Health Care Data Ecosystems: Taxonomy Development Study

**DOI:** 10.2196/63396

**Published:** 2025-02-18

**Authors:** Simon Scheider, Mostafa Kamal Mallick

**Affiliations:** 1 Fraunhofer Institute for Software and Systems Engineering Dortmund Germany

**Keywords:** data catalogs, data ecosystems, findability, accessibility, interoperability, and reusability, FAIR, health care, metadata, taxonomy

## Abstract

**Background:**

In the European health care industry, recent years have seen increasing investments in data ecosystems to “FAIRify” and capitalize the ever-rising amount of health data. Within such networks, health metadata catalogs (HMDCs) assume a key function as they enable data allocation, sharing, and use practices. By design, HMDCs orchestrate health information for the purpose of findability, accessibility, interoperability, and reusability (FAIR). However, despite various European initiatives pushing health care data ecosystems forward, actionable design knowledge about HMDCs is scarce. This impedes both their effective development in practice and their scientific exploration, causing huge unused innovation potential of health data.

**Objective:**

This study aims to explore the structural design elements of HMDCs, classifying them alongside empirically reasonable dimensions and characteristics. In doing so, the development of HMDCs in practice is facilitated while also closing a crucial gap in theory (ie, the literature about actionable HMDC design knowledge).

**Methods:**

We applied a rigorous methodology for taxonomy building following well-known and established guidelines from the domain of information systems. Within this methodological framework, inductive and deductive research methods were applied to iteratively design and evaluate the evolving set of HMDC dimensions and characteristics. Specifically, a systematic literature review was conducted to identify and analyze 38 articles, while a multicase study was conducted to examine 17 HMDCs from practice. These findings were evaluated and refined in 2 extensive focus group sessions by 7 interdisciplinary experts with deep knowledge about HMDCs.

**Results:**

The artifact generated by the study is an iteratively conceptualized and empirically grounded taxonomy with elaborate explanations. It proposes 20 dimensions encompassing 101 characteristics alongside which FAIR HMDCs can be structured and classified. The taxonomy describes basic design characteristics that need to be considered to implement FAIR HMDCs effectively. A major finding was that a particular focus in developing HMDCs is on the design of their published dataset offerings (ie, their metadata assets) as well as on data security and governance. The taxonomy is evaluated against the background of 4 use cases, which were cocreated with experts. These illustrative scenarios add depth and context to the taxonomy as they underline its relevance and applicability in real-world settings.

**Conclusions:**

The findings contribute fundamental, yet actionable, design knowledge for building HMDCs in European health care data ecosystems. They provide guidance for health care practitioners, while allowing both scientists and policy makers to navigate through this evolving research field and anchor their work. Therefore, this study closes the research gap outlined earlier, which has prevailed in theory and practice.

## Introduction

### Challenges of Health Care Systems

In the 21st century, health care systems worldwide are experiencing a tremendous increase in data, driven by advances in medical technology, digital health records, and wearable devices [1]. This flood of data holds immense potential for data-driven health innovations, building upon large-scale real-world data (RWD) and real-world evidence (RWE) [2]. However, health care systems face multiple challenges that hinder the effective use of RWD to generate RWE and thus data-driven health innovations. One primary issue is the integration of heterogeneous datasets [3]. RWD stem from diverse sources, such as electronic health records, imaging, and genomic data, frequently exhibiting incompatible or unknown data formats, which complicates harmonization, particularly across different entities [4,5]. Furthermore, finding and accessing suitable RWD represents a hurdle for medical research due to their origins from disparate patient populations, health care systems, and data collection methodologies [6]. This impairs the effective discovery of and access to a sufficient number of both available and adequate datasets. Moreover, even if enough RWD are discovered and access is established, another challenge lies in ensuring scientific rigor and reproducibility of generated RWE (ie, medical studies) that becomes increasingly difficult in today’s data-intensive health research [7]. Medical studies constantly require larger, high-quality datasets to generate meaningful and reproducible RWE [5,6,8]. However, unknown RWD management practices threaten study reliability, while making the validation of results (ie, RWE) across studies difficult [2,7]. Besides that, the diversity of national health care systems increases the prevailing differences in health care data infrastructures across countries that, in turn, lead to additional barriers for organizations to share and use RWD at a large scale [9]. Moreover, health care systems must navigate complex legal requirements with regard to sharing and processing RWD [8,9]. For instance, the European jurisdiction mandates strict data governance and security standards that, while essential, impair data-driven health innovations in the absence of adequate data-sharing infrastructures [9]. As a result, RWD are fragmented and isolated within single organizations, whereby data sharing and use are limited. Because of all these challenges, the rapidly increasing amount of RWD cannot be harnessed to its full potential for producing health care innovations (ie, RWE).

### Metadata Catalogs as a Promising Solution

Against this background, data ecosystems as technical and organizational infrastructures within the healthcare sector represent auspicious solutions, that is, *health care data ecosystems* (amplified in, eg, the studies by Lovestone and EMIF Consortium [10], Manogaran et al [11], and Sharon and Lucivero [12]). These evolving networks enable legally compliant use of RWD [13]. Their key function is market mechanisms instantiated by metadata catalogs [5,14] that define and describe the intricate web of RWD circulating between a potentially arbitrary number of actors in the ecosystem [15]. Hence, such *health metadata catalogs* (HMDCs) are crucial components of modern health care data ecosystems, for example, EHDEN (European Health Data and Evidence Network), EHDS2 (European Health Data Space 2), Elixir, EUCAIM (European Federation for Cancer Images), IDERHA (Integration of Heterogeneous Data and Evidence towards Regulatory and Health Technology Assessment Acceptance), and Gaia-X. Multimedia Appendix 1 provides a comprehensive overview about the most important European Union (EU) initiatives. For example, EHDS2 aims at creating a European infrastructure for the secure exchange and secondary use of health data across EU member states [16]. Therein, HMDCs belong to the core infrastructure services to enable standardized organization of and controlled access to RWD for research. The pilot infrastructure of the EHDS is implemented by the HealthData@EU initiative.

Since HMDCs provide an effective method for systematically sharing and using RWD within data ecosystems [3], they potentially allow harnessing crucial benefits corresponding to the challenges outlined earlier. First, HMDCs facilitate integrating heterogenous datasets across health care systems. They help to transcend diverse data types, which eases the integration, standardization, and harmonization of data within data ecosystems [3,5]. This is essential for medical research that requires huge pools of accessible RWD appropriate for their investigations [7]. Second, as finding and accessing adequate RWD effectively is vital for medical research [6], HMDCs entail added value by offering a governed data search and access framework embedded into the technical infrastructures of the underlying ecosystems [17]. They provide a tool for data discovery to precisely characterize, locate, and filter RWD on the basis of a myriad of factors [5,17]. Third, HMDCs support transparent and reproducible research processes by helping scientists in replicating studies and validating their results [18]. Such transparency is fundamental for building trust in the reliability and validity of RWE [2]. Finally, HMDCs facilitate data-intensive research, generally, as they establish unified health care data infrastructures for allocating, accessing, and using RWD of connected data providers [19]. In doing so, they reduce barriers for organizations to integrate their otherwise isolated RWD within data ecosystems. At the same time, HMDCs bridge prevailing differences between national health care systems and retain full control of data providers [5,19]. To this end, they establish robust data security and governance frameworks that are aligned to the applicable jurisdictions [5,15].

As a result, HMDCs represent an auspicious medium against the fragmentation and isolation of RWD [5]. However, since HMDCs are novel constructs, typically in premature phases [20], their ascribed benefits are primarily backed by the literature rather than evidence from practice. Nevertheless, HMDCs are likely to provide means for using RWD systematically within and across health care data ecosystems, potentially resulting in more efficient RWE generation.

In Europe, HMDCs are of particular importance because the EU health care sector exhibits a broad diversity across member states, all with their own health care systems and policies. Consequently, there is a need to focus heavily on standardizing and harmonizing both data and metadata across different countries for facilitating data sharing and legally compliant data use [21]. More specifically, the diversity of national health care systems [22], the restrictiveness of data protection regulations [8,9], and the fragmentation and isolation of health data [23] make operative health care data ecosystems and HMDCs a paramount concern for the European health care industry. Therefore, this study adopts a European focus.

### Theoretical Background

Originally, data catalogs are organized collections of datasets that provide descriptive information within an organization [24,25]. They act as centralized repositories, making it easier for data consumers to discover, understand, and access the information they need [26]. Enterprise data management platforms often comprise such centralized data catalogs implying storage of data within their peripheries [25,27]. If data are not encapsulated within the organization but integrated into decentral or federated networks [28], the literature commonly refers to such environments as data ecosystems with metadata catalogs as key function [5,14]. This study considers metadata catalogs as decentralized or federated constructs that are mutually exclusive to centralized ones. For simplification, only the term decentralized is used.

Metadata describe dataset attributes, such as source, format, structure, provenance, owner, access, or governance modalities [29]. Metadata catalogs act as “catalogues of data catalogues,” dedicated to enhancing discoverability, usability, and management of distributed datasets [30]. Within data ecosystems, metadata catalogs are a mechanism that provides a standardized way for recording, disclosing, and making available information about all relevant kinds of phenotypes describing datasets, while ensuring legally compliant access and sharing practices [3,14]. If these datasets are health data, it is henceforth referred to such constructs as *HMDCs*. Consequently, HMDCs manage heterogenous health information integrated into health care data ecosystems [5]. They ensure that these diverse and highly sensitive datasets are effectively organized and understood [4], while facilitating their systematic use [3,5,14]. This requires dedicated, yet unknown, design elements to be unveiled by the study.

### Research Gap, Objective, and Questions

After having clarified the added value of HMDCs, the research gap is demarcated by reviewing related work. Therefrom, the research problem is identified, which leads to the research objectives. These objectives then allow to derive the research questions, required to define a meaningful research methodology.

To begin with, Labadie et al [24] foster the understanding of data catalogs by classifying corresponding initiatives. The authors propose a taxonomy for data catalogs and present 3 case studies. However, similar to Ehrlinger et al [26] and Jahnke and Otto [25], Labadie et al [24] focus on intraorganizational data sharing using centralized catalogs. Moreover, they neglected health use cases. Remy et al [3] conducted a design science study to build an integrated catalog for health research metadata. The artifact enables medical scientists to analyze phenomena that require a view across several domains. The authors are among the first who provide design knowledge usable in HMDC contexts. Although, similar to the findings of the previously presented literature sources, Remy et al [3] accentuate centralized catalogs. Almeida et al [19] present a platform that provides a set of tools, compliant with the findability, accessibility, interoperability, and reusability (FAIR) principles, to help data holders sharing biomedical databases while allowing data consumers to discover and apply for them. However, the authors only consider a narrow use case instead of generating universally applicable design knowledge. Similarly, Oliveira et al [15] developed a holistic stakeholder agnostic catalog framework for biomedical datasets. Researchers can explore metadata held decentralized at federated nodes, with distinct levels of granularity being conceivable. Extending this initial design knowledge specific to biomedical data, Swertz et al [5] proposed a unified framework for sharing health data across catalogs. It encompasses multiple centralized and decentralized catalogs. The authors offer recommendations to establish an integrated community as an open catalog ecosystem. This theoretical basis for HMDCs builds upon and is enriched by similar research. Specifically, Bergeron et al [17] developed a catalog toolkit to support creating comprehensive as well as user- and study-friendly HMDCs. Almeida and Oliveira [30] produced a framework to simplify the process of building an HMDC for exposing metadata, while providing analysis capacities. Apparently, there is a tendency from centralized to decentralized data catalogs in health care. However, for HMDCs, a *research gap* prevails concerning (1) empirically grounded and actionable design knowledge that is (2) universally applicable to (3) the broad array of use cases and EU initiatives associated with health care data ecosystems.

In general, the generation of design knowledge about an artifact is crucial as it provides the intellectual foundation to advance the respective body of scientific knowledge, while facilitating development efforts in practice [31]. In particular, HMDC design knowledge can harmonize and sustain the multitude of different EU initiatives by following a systematic approach to problem-solving [31,32]. Therefore, its generation must adhere to a rigor design process [33,34]. This process must ensure empirical grounding for the sake of efficiency, effectiveness, and quality assurance, which inevitably favors quality, adaptiveness, and impact of the generated results [35]. Likewise, the prevailing lack of design knowledge causes difficulties concerning the adoption and use of HMDCs in practice and theory, revealing the *research problem*. To remedy this problem, the *research objective* is to provide actionable design knowledge that is universally applicable in real-world HMDC use cases, thus allowing to infer the following *research questions* (RQs):

- RQ1:
  What are taxonomy elements (ie, dimensions and characteristics) to structure HMDCs from a design science perspective?
- RQ2:
  How does the proposed taxonomy effect real-world use cases?

According to Hevner et al [33], a *design science* perspective means to examine and create information system (IS) artifacts to solve practical problems. A taxonomy is a suitable approach to address RQ1 because it provides a set of elementary building blocks and prescriptions for effectively designing such artifacts [36,37]. It targets a broad and diverse audience, including health care IS engineers and architects, health data holders and scientists, health care economists and researchers, as well as legal and ethical regulatory bodies, while accentuating the European health care sector.

## Methods

### Overview

**Figure 1:**
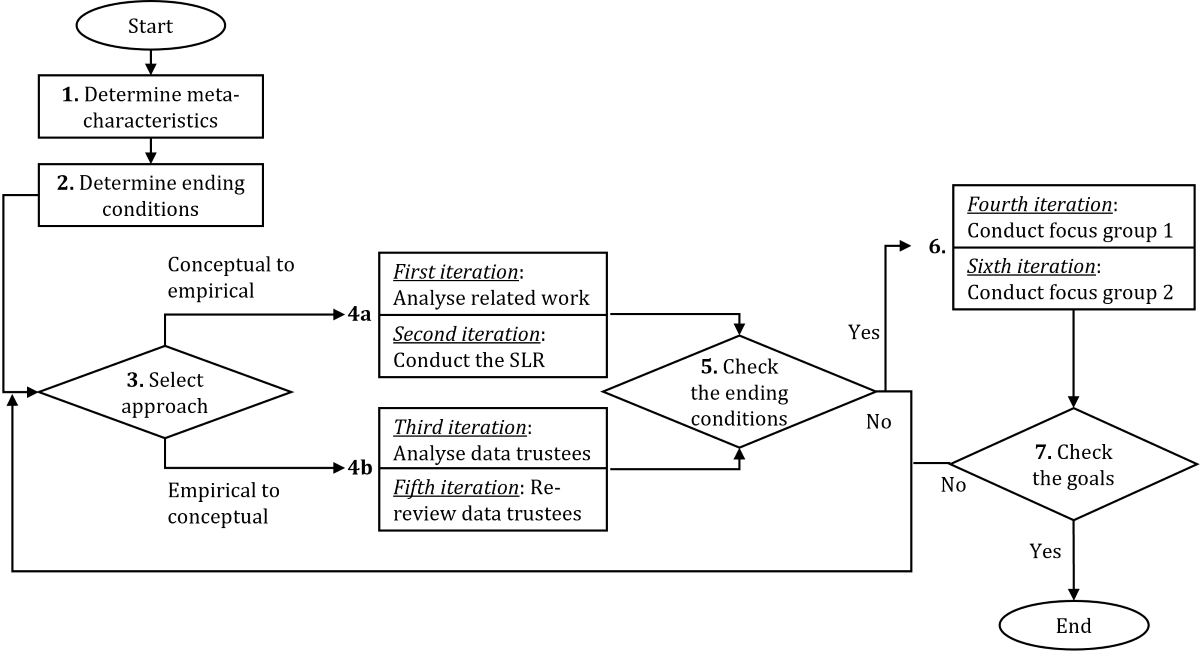
Applied research method of the taxonomy development study based on Nickerson et al [37] and Kundisch et al [36].

Taxonomies are common approaches in IS research to classify, understand, and examine complex issues [38]. For their development, the method of Nickerson et al [37] is applied to identify dimensions and characteristics of HMDCs. The authors propose generating knowledge conceptually (eg, from the literature) and empirically (eg, analyzing objects of interest). This approach is referred to as the gold standard to build taxonomies in IS research [36]. As refinement, the methodological update of Kundisch et al [36] is incorporated, adding an evaluation process by means of focus groups. The authors’ refinement enhances the assessment of value created by the taxonomy [39]. Corresponding to these 2 methods, the research design is divided into the 7 steps shown in Figure 1 based on the studies by Nickerson et al [37] and Kundisch et al [36]. The numbers 1 to 7 represent methodological steps explained in the following sections.

In step 1 in Figure 1, a *meta-characteristic* is specified in orientation towards the taxonomy’s purpose so that each subordinated characteristic and dimension follows from it. On the basis of RQ1, the meta-characteristic was defined as “distinguishing key design elements of HMDCs.” It facilitates selecting meta-dimensions as well as inferring characteristics and classifying them to dimensions. To define the meta-dimensions, the FAIR framework was used [7]. These well-known data principles postulate an accepted approach to the discoverability and usability of RWD [19]. While FAIR emphasizes making data interoperable and reusable, it inherently involves considerations related to data governance and harmonization [40].

In step 2 in Figure 1, *ending conditions* for the iterative part of the process are defined, determining its termination criteria. The ending conditions were chosen on the basis of Nickerson et al [37] and Scheider et al [41] in terms of subjective and objective criteria. Ultimately, 6 design iterations were required until all conditions listed in Table 1 were fulfilled.

**Table 1:** Ending conditions for taxonomy development adopted from Nickerson et al [37].

Ending conditions			Design iterations														
			1		2		3		4		5		6				
**Objective**																	
	All papers were examined.					✓		✓		✓		✓					
	No object was merged with another or split.					✓				✓		✓					
	Each characteristic is classified by one object.	✓		✓		✓		✓		✓		✓					
	No new dimensions or characteristics were added.					✓				✓		✓					
	Dimensions or characteristics were neither merged nor split.					✓				✓		✓					
	Each dimension is unique and not duplicated.	✓		✓		✓		✓		✓		✓					
	Every characteristic is unique within its dimension.	✓		✓		✓		✓		✓		✓					
	Each cell is unique and not repeated.	✓		✓		✓		✓		✓		✓					
**Subjective**																	
	Conciseness: no unnecessary dimensions and characteristics					✓		✓		✓		✓					
	Robust: dimensions and characteristics differentiate objects					✓		✓		✓		✓					
	Comprehensiveness: all objects can be classified					✓		✓		✓		✓					
	Extension: dimensions and characteristics can be added easily			✓		✓		✓		✓		✓					
	Explanatory: dimensions and characteristics describe all objects					✓				✓		✓					

In steps 3 to 5 in Figure 1, we repeatedly chose between either an inductive or a deductive path. The former is a conceptual-to-empirical attempt (step 4a in Figure 1) to infer dimensions and characteristics from theory. The latter reflects an empirical-to-conceptual procedure (E2C; step 4b in Figure 1) to derive characteristics from real-world analysis objects and to classify them in dimensions. After each iteration (ie, steps 3 to 5 in Figure 1), the ending conditions are checked (ie, step 5 in Figure 1). If all ending conditions are fulfilled, an evaluation step (ie, step 6 in Figure 1) follows, integrated by focus groups [36]. In case the focus group iteration does not imply changes to the taxonomy (ie, step 7 in Figure 1), the artifact is finished and the methodological process terminates. After 5 design iterations (ie, 4 times of executing steps 4a and 4b and executing step 6 once in Figure 1), all ending conditions were fulfilled (ie, step 5 in Figure 1) and the subsequent focus group did not result in any major changes (ie, step 7 in Figure 1). Thus, the taxonomy was completed [36]. Because 6 design iterations were traversed and it was ensured that the focus group experts covered all dimensions relevant for HMDCs, the taxonomy achieved result saturation.

To ensure transparency of the taxonomy development process, Multimedia Appendix 2 shows intermediary taxonomies after certain iterations. Furthermore, it offers a table linking key references from the inductive (ie, the literature) and deductive iterations (ie, analysis objects) to the dimensions of the final taxonomy [5,10,14,16,17,19,24,28,30,41-78].

### Inductive Design Iterations for Taxonomy Development

In the first iteration, an initial set of dimensions and characteristics was derived from former research (ie, step 4a in Figure 1), consolidating the related work addressed in the Introduction section.

In the second iteration, a structured literature review (SLR) was carried out (ie, step 4a in Figure 1) [36]. The method of Kitchenham et al [79] was applied (ie, 1-6), while orienting toward its application in the study by Scheider et al [41]. At the outset, RQ1 was adopted as the (1) research question guiding the SLR. The (2) search process comprised HMDC-related conference and journal papers. The search string was defined as (ALL (health AND data AND catalog) OR ALL (health AND metadata AND catalog) AND ALL (data AND catalog AND technologies)). Primarily, Scopus and IEEE Xplore were used, and the operands were deployed on documents’ titles, abstracts, and authors’ keywords. The 2 databanks were leveraged due to their multidisciplinary nature covering research in all fields relevant for HMDCs. Following Scheider et al [41], (3) inclusion and exclusion criteria were created to identify and filter papers. First, the literature not available in English was excluded. Second, inaccessible papers were removed. Third, each paper retrieved was reviewed by 2 researchers for whether it covers HMDCs in the broader sense. This means that papers had to address a design perspective, as defined by Hevner et al [33]. Articles were emphasized that dealt with “patient-related” data, while ones about aggregated health data (eg, regions and countries) were neglected. The same holds true for catalogs about health-oriented surveys and analysis results (eg, studies). Due to broadly formulated keywords in the search string, initially retrieved literature contained many papers outside the thematical scope. To this end, the third inclusion or exclusion criterion was examined by screening titles and abstracts before reviewing the entire content of the papers. Since 2 researchers constantly worked together in (3), one can argue for reliable objectivity in paper selection.

**Figure 2:**
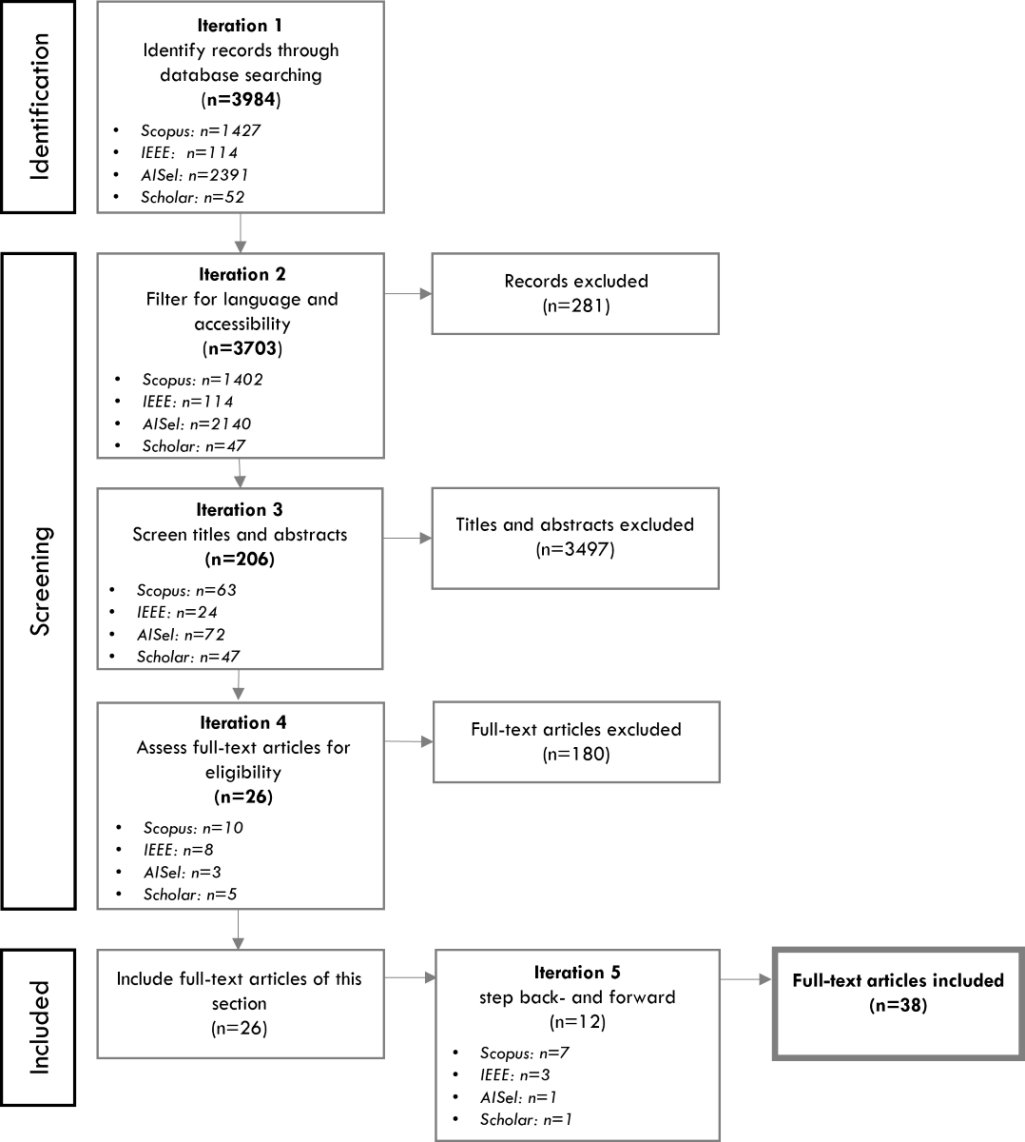
Structured literature review statistics presented in the PRISMA (Preferred Reporting Items for Systematic Reviews and Meta-Analyses) flowchart.

Building upon the inclusion and exclusion criteria, the initial (4) data collection resulted in 18 papers in the Scopus and IEEE Xplore search (iteration 4 in Figure 2). Subsequently, backward (ie, referenced articles) and forward (ie, citing articles) stepping was conducted [80], which added 12 articles. Figure 2 shows the SLR statistics expressed by a PRISMA (Preferred Reporting Items for Systematic Reviews and Meta-Analyses) flowchart.

The SLR was expanded by a search via Google (Google Search) and the AISeL database for extension and verification. The Google search engine served to identify whitepapers using a consolidated search string compared to (2). For AISeL, the same steps executed in Scopus and IEEE Xplore were applied (ie, 2 and 3), except that the research team looked for the search terms in titles only to keep the number of results feasible. Once duplicates were removed, Google and AISeL added 8 papers to the literature collection. To test theoretical saturation [81], “quick searches” were carried out in other databases (eg, ACM) checking whether the top results, first, match the inclusion and exclusion criteria and, second, are not already in the collection. Since these quick searches did not add new papers, the literature collection was considered representative [41]. Excluding related work of the first iteration, the collection comprised 38 publications, of which the most important items are listed in Table 2.

**Table 2:** List of most important literature from the SLR^a^ used for taxonomy development.

Study		Year			Title
**Top 10 academic papers**					
	Alvarellos et al [42]	2023		Democratizing clinical-genomic data: How federated platforms can promote benefits sharing in genomics	
	Almeida and Oliveira [30]	2024		MONTRA2^b^: A web platform for profiling distributed databases in the health domain	
	Almeida et al [19]	2023		A FAIR^c^ approach to real-world health data management and analysis	
	Bergeron et al [17]	2018		Fostering population-based cohort data discovery: The Maelstrom Research cataloguing toolkit	
	Scheider et al [41]	2023		Exploring design elements of personal data markets	
	Ehrlinger et al [26]	2021		Data catalogs: a systematic literature review and guidelines to implementation	
	Labadie et al [24]	2020		Fair enough? enhancing the usage of enterprise data with data catalogs	
	Lovestone and EMIF Consortium [10]	2020		The European medical information framework: a novel ecosystem for sharing health care data across Europe	
	Oliveira et al [15]	2019		EMIF^d^ Catalogue: a collaborative platform for sharing and reusing biomedical data	
	Swertz et al [5]	2022		Towards an Interoperable Ecosystem of Research Cohort and Real-world Data Catalogues Enabling Multi-center Studies	
**Top 5 nonacademic papers**					
	European Medicines Agency [43]		2022	Good Practice Guide for the use of the Metadata Catalogue of Real-World Sources	
	European Medicines Agency [44]		2022	List of metadata for Real World Data catalogues	
	Directorate-General for Health and Food Safety [82]		2022	The European Health Data Space	
	Jahnke and Otto [25]		2022	Data Catalogs - Implementing Capabilities for Data Curation, Data Enablement and Regulatory Compliance	
	TEHDAS^e^ [16]		2022	EHDS^f^ Semantic interoperability framework	

^a^SLR: structured literature review.

^b^MONTRA2: Modular Next-generation Research Analysis.

^c^FAIR: findability, accessibility, interoperability, and reusability.

^d^EMIF: European Medical Information Framework Catalogue.

^e^TEHDAS: Towards European Health Data Space.

^f^EHDS: European Health Data Space.

Throughout the steps (2) to (4), a (5) quality assessment step was integrated on the basis of the criteria suggested by Kitchenham et al [79], that is, inclusion or exclusion criteria, relevant article coverage, literature corpus assessment, and study descriptions.

**Figure 3:**
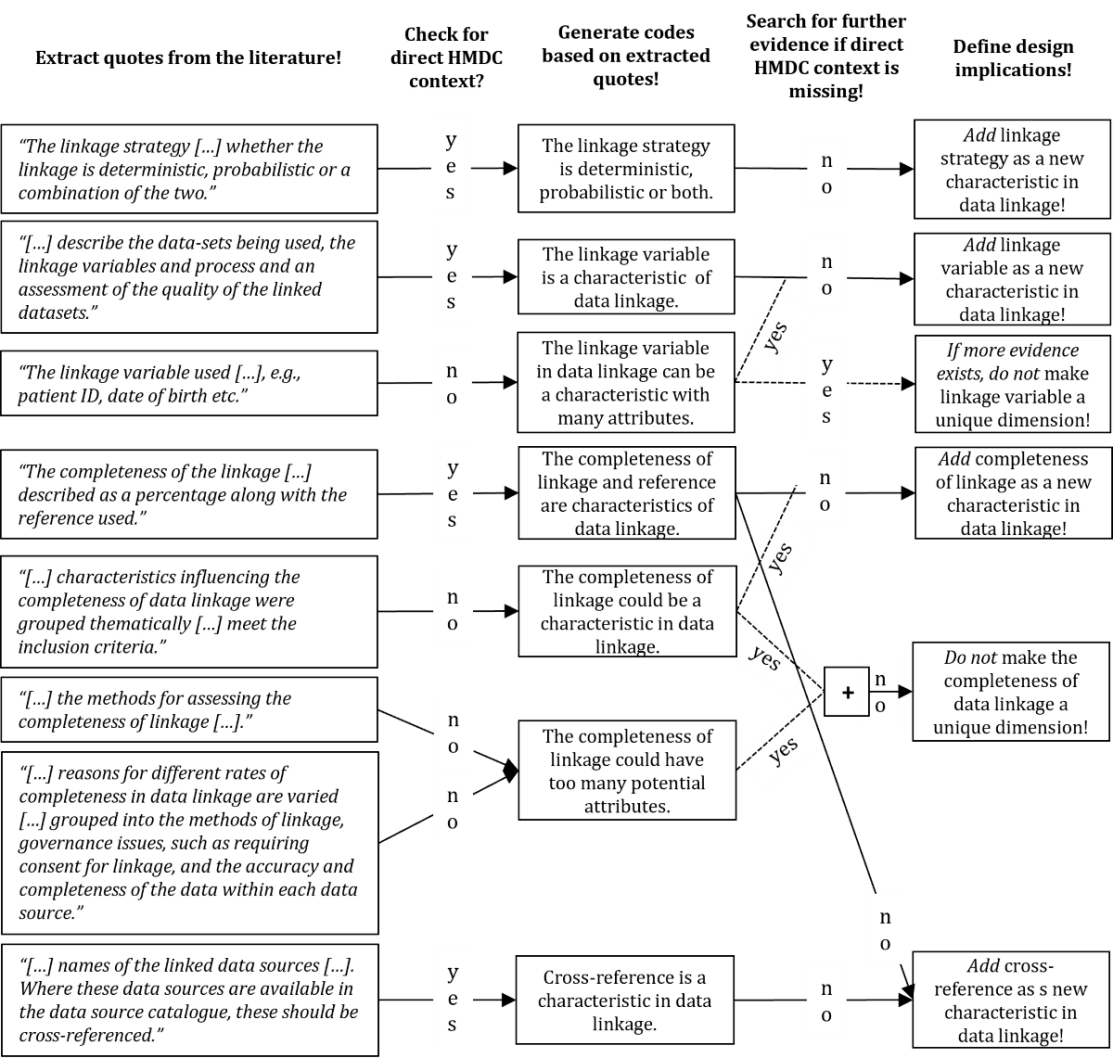
Examples for literature coding for inductive taxonomy development.

For (6) data analysis, phrases (“quotes”) from articles with useful content for HMDC designs were extracted. Following the approaches of Saldana [83] and Pratt [84], those phrases were coded, inserted in a tabular structure, and iteratively generalized. As in steps (3) and (9), two researchers analyzed the literature to reduce subjectivity biases. Figure 3 shows how quote extractions relating to the dimension of data linking are coded and design implications are derived. Particularly, whenever there was a direct connection to an HMDC context, quotes became design implications immediately, for example, linkage strategy (first quote in Figure 3). If a direct connection was missing (eg, linkage variable as a new characteristic; third quote in Figure 3), more evidence was required to transform codes into design implications (ie, second quote in Figure 3). Finally, considering the influential factors proposed by Mwita [81] (eg, study purpose, research design, sample size variability, and analysis approach), data saturation in the SLR is likely.

### Deductive Design Iterations for Taxonomy Development

Applying the E2C approach (ie, step 4b Figure 1) in the third and fifth iterations, health data catalogs from practice were initially listed, as identified in the first 2 iterations. This set was extended by a Google search to identify analysis objects not encountered in inductive iterations. The research team searched for analysis objects using the browser’s incognito mode to circumnavigate carryover effects from previous searches [85]. The keywords from the SLR were used as an orientation to avoid limiting the results unconsciously [41]. Analysis objects were excluded if meaningful information could not be obtained. This characteristic was defined as access to analyzable information describing the analysis object, that is, data retrievable either from websites or demo applications [85]. Analysis objects were also excluded if information was meaningful but unavailable in German or English. However, metadata catalogs under construction were not excluded per se [41]. The set of analysis objects was created in the first quarter of 2024. The final analysis objects are listed in Table 3.

**Table 3:** Health metadata catalogs from practice used for taxonomy development.

HMDCs^a^	Classification	Status
BBMRI-ERIC^b^ Data Directory [45]	Decentral	Operative
Catalogue of Mental Health Measures [46]	Central	Operative
Compendium Data Catalog for Healthcare [47]	Central	Operative
EHDEN^c^ Portal [48]	Decentral	Operative
Elixir (BioSamples [86] and FAIRsharing [87])	Decentral	Operative
EMIF^d^ Data Catalogue [49]	Decentral	In progress
EUCAIM^e^ Cancer Image Europe [50]	Decentral	Operative
European Health Information Portal [51]	Central	Operative
Fjelltopp Data Catalogues for Health [52]	Central	Operative
HealthRI^f^ Data Catalogues [53]	Decentral	Operative
Helsedata Explore Data Sources [54]	Decentral	Operative
IDERHA^g^ Metadata Catalogue (no public access)	Decentral	In progress
IHME^h^ Global Health Data Exchange [88]	Central	Operative
IQVIA^i^ Health Data Catalogue [55]	Central	Operative
Kraken Health Data Pilot [56]	Decentral	In progress
Lifebit Precision Medicine Data Catalogue [57]	Decentral	Operative
MACH^j^ Clinical and Research Data Catalogue [89]	Decentral	Operative
Maelstrom Research Data Catalogue [58]	Decentral	Operative
Yoda Trials Data Catalogue [59]	Decentral	In progress

^a^HMDC: health metadata catalog.

^b^BBMRI-ERIC: Biobanking and Biomolecular Resources Research Infrastructure – European Research Infrastructure Consortium.

^c^EHDEN: European Health Data and Evidence Network.

^d^EMIF: European Medical Information Framework.

^e^EUCAIM: European Federation for Cancer Images.

^f^HealthRI: Health Research Infrastructure.

^g^IDERHA: Integration of Heterogeneous Data and Evidence towards Regulatory and Health Technology Assessment Acceptance.

^h^IHME: Institute for Health Metrics and Evaluation.

^i^IQVIA: Information, Quality, Value, Innovation, and Access.

^j^MACH: Melbourne Academic Centre for Health.

The catalogs were examined by classifying them alongside the design elements of the preliminary taxonomy (Multimedia Appendix 2). We tried to assign a catalog to a single characteristic in each dimension. This deductive activity was conducted by 2 researchers, whereby three cases could occur [41]: (1) on the basis of available information, the analysis object could be assigned to an existing characteristic in a dimension; (2) due to a lack of available information, the analysis object could not be assigned to any characteristic in a dimension; and (3) the analysis object contained information about a dimension but could not be associated with a characteristic defined therein. Evidently, the third case led to modifications of a current set of taxonomy design elements. During the third iteration, the occurrence of this case decreased continuously until only the first and second ones appeared. Because the ending conditions were fulfilled at that time, the taxonomy-building process was terminated and the first focus group session started. Alterations were proposed by focus group members in this session. This led to another E2C approach as the fifth iteration in which specific design elements were emphasized, as addressed by the focus group experts. The corresponding analysis procedure (ie, 1-3) remained the same as in the third iteration, while examining decentral analysis objects only (Table 3).

### Evaluative Design Iterations for Taxonomy Development and Use Case Cocreation

Following Figure 1, an evaluation by focus groups is needed after the fulfillment of all ending conditions (ie, in step 6). Focus groups help gather more data than individual interviews, since experts respond to the input of others, triggering discussions and idea generation [23]. According to Szopinski et al [39], focus groups are particularly suitable to assess the comprehensiveness, robustness, understandability, and extensibility of a taxonomy, as well as the shape of dimensions and characteristics. Members were recruited on the basis of the target audience of the taxonomy (see section about Research gap, objective, and questions). We ensured that they have substantial knowledge with regard to HMDCs, stemming from EU initiatives (Multimedia Appendix 1). Ultimately, the focus group consisted of 7 experts. However, scheduling conflicts affected the organization of meetings. The first focus group had to be split into 2 sessions, involving 5 experts. The second focus group iteration required 3 separate sessions to accommodate all 7 experts. Multimedia Appendix 3 shows the distribution of experts to sessions per iteration. Focus group sessions occurred in the fourth and sixth iteration. Their rough agenda was as follows: (1) the objective of the taxonomy (only fourth iteration) and current version (fourth and sixth iteration) were presented; (2) a dimension with its characteristics was explained; (3) experts shared their expectations about this dimension, especially with regard to real-world use cases; and (4) we triggered discussions by contrasting expectations. Because the focus groups served for evaluation purposes and concrete design elements were discussed in the plenum directly, we dispensed with detailed coding. This lightweight effort is inspired and justified by similar research studies published in high-ranking IS journals [14,41]. Therefore, we argue that our approach successfully mitigates any subjectivity biases because specific taxonomy design elements were addressed and discussed in the focus groups directly. This narrow focus led to the derivation of tangible design activities (ie, deletion, addition, alteration, and renaming of design elements and cocreation of use cases).

In the fourth iteration, the focus group resulted in substantial changes of dimensions and characteristics, particularly regarding taxonomy elements of data accessibility and findability (Multimedia Appendix 2). On the contrary, the second session in the sixth iteration caused minor adjustments only (eg, renaming a few characteristics), emphasizing the cocreation of use cases. Finally, the third session of the sixth iteration merely led to refined use case formulations and merging of content. Given these small adjustments, a sufficient saturation in results was considered [23] and the taxonomy development was terminated (ie, step 7). Multimedia Appendix 3 lists the focus group members who participated in the evaluation of the taxonomy and the cocreation of use cases during the fourth and sixth iterations. It further states the EU initiatives the experts were recruited from and whether these initiatives have an operative HMDC or one that is still under development.

### Ethical Considerations

In the run-up to the sessions, the experts received an information sheet explaining the study context, the procedure, and the approach to gathering and processing interview data. It was assured that no personal information will be disclosed. Experts were informed about their right to opt out. During the focus group sessions, only anonymous interview data in the form of handwritten notes were collected. Neither a video or voice recording nor the transcription of interview material was used. Experts did not receive any financial compensation. Hence, conducting focus groups did not require the official approval of an ethics review committee.

## Results

### Taxonomy for FAIR HMDCs

#### Overview

Table 4 shows the taxonomy containing 20 dimensions (*D_n_*) and 101 characteristics (*C_nm_*) structured alongside the FAIR data principles as meta-dimensions [40]. For visualization, morphologies were considered (Multimedia Appendix 2) as they demonstrate the structure and arrangement of taxonomy elements [90]. Importantly, the meta-dimension of data accessibility has exclusive dimensions pertaining to the general design of HMDCs. The other meta-dimensions relate to design elements of metadata assets published *within* the HMDC and the data accessibility constraints. On the basis of the study by Nickerson et al [37], nonexclusiveness was chosen for some of the dimensions associated with metadata assets. The reason is that they propose structural characteristics, which might be accumulated for creating effective metadata assets in an HMDC. Exclusivity of dimensions allows to categorize design elements into mutually distinct characteristics, ensuring clarity and avoiding overlaps. Nonexclusivity can accommodate complex multidimensional relationships by enabling design elements belonging to multiple characteristics [37]. Correspondingly, the difference between OR and XOR in the middle column of Table 4 is that OR is used for nonexclusive characteristics, while XOR is used for exclusive ones.

**Table 4:** Taxonomy of FAIR^a^ HMDCs^b^ presented as a morphological box.

Dimension (D_n_)		Characteristics (C_nm_)		Exclusive or nonexclusive
**Findability**				
	*D*_*1*_: data source	*C*_*1.1*_: patients’ health OR *C*_*1.2*_: medical procedures OR *C*_*1.3*_: medical products OR *C*_*1.4*_: others	Nonexclusive	
	*D*_*2*_: managerial. details	*C*_*2.1*_: holder OR *C*_*2.2*_: origin OR *C*_*2.3*_: collection OR *C*_*2.4*_: qualification OR *C*_*2.5*_: financials OR *C*_*2.6*_: others	Nonexclusive	
	*D*_*3*_: data type	*C*_*3.1*_: admin XOR *C*_*3.2*_: primary care XOR *C*_*3.3*_: secondary care XOR *C*_*3.4*_: registries XOR *C*_*3.5*_: others	Exclusive	
	*D*_*4*_: population information	*C*_*4.1*_: disease OR *C*_*4.2*_: family linkages OR *C*_*4.3*_: lifestyle factors OR *C*_*4.4*_: population OR *C*_*4.5*_: sociodemographic OR *C*_*4.6*_: catchment area coverage	Nonexclusive	
	*D*_*5*_: data sensitivity	*C*_*5.1*_: synthetic data XOR *C*_*5.2*_: anonymized data XOR *C*_*5.3*_: pseudonymized data XOR *C*_*5.4*_: personal data	Exclusive	
**Accessibility**				
	*D*_*6*_: catalogue accessibility	*C*_*6.1*_: public XOR *C*_*6.2*_: hybrid XOR *C*_*6.3*_: private	Exclusive	
	*D*_*7*_: dataset accessibility	*C*_*7.1*_: free XOR *C*_*7.2*_: formal request XOR *C*_*7.3*_: strictly limited XOR *C*_*7.4*_: others	Exclusive	
	*D*_*8*_: access control	*C*_*8.1*_: catalog operator OR *C*_*8.2*_: internal DAC^a^ OR C_*8.3*_: external DAC OR C_*8.4*_: none OR *C*_*8.5*_: others	Nonexclusive	
**Interoperability**				
	*D*_*9*_: program discoverability	*C*_*9.1*_: Beacon OR *C*_*9.2*_: BBMRI-MIABIS^d^ OR *C*_*9.3*_: bioimage OR *C*_*9.4*_: CESSDA^e^ OR *C*_*9.5*_: DCAT^f^ OR *C*_*9.6*_: ECRIN-CRMDR^g^ OR C_*9.7*_: FairShairing OR *C*_*9.8*_: INSPIRE^h^ OR *C*_*9.9*_: PHIRI^i^ OR *C*_*9.10*_: others	Nonexclusive	
	*D*_*10*_: semantic interoperability	*C*_*10.1*_: CDISC-SDTM^j^ OR *C*_*10.2*_: LOINC^k^ OR *C*_*10.3*_: OMOP^l^ OR *C*_*10.4*_: Oorphanet standards OR *C*_*10.5*_: SNOMED^m^ OR *C*_*10.6*_: others	Nonexclusive	
	*D*_*11*_: interoperable communication	*C*_*11.1*_: DICOM^n^ OR *C*_*11.2*_: HL7 FHIR^o^ OR *C*_*11.3*_: IDMP^p^ OR *C*_*11.4*_: ISO 800-110^q^ OR *C*_*11.5*_: others	Nonexclusive	
	*D*_*12*_: CDM^r^	*C*_*12.1*_: type OR *C*_*12.2*_: reference OR *C*_*12.3*_: release frequency	Nonexclusive	
	*D*_*13*_: ETL^s^ status	*C*_*13.1*_: planned XOR *C*_*13.2*_: in progress XOR *C*_*13.3*_: completed	Exclusive	
	*D*_*14*_: vocabularies	*C*_*14.1*_: medicinal product OR *C*_*14.2*_: cause of death OR *C*_*14.3*_: quality of life measurement OR *C*_*14.4*_: prescription OR *C*_*14.5*_: dispensing OR *C*_*14.6*_: indication OR *C*_*14.7*_: procedures OR *C*_*14.8*_: genetic data OR *C*_*14.9*_: biomarker data OR *C*_*14.10*_: medical event	Nonexclusive	
**Reusability**				
	*D*_*15*_: collection methodology	*C*_*15.1*_: collection governance OR *C*_*15.2*_: collection process OR *C*_*15.3*_: dataset updates OR *C*_*15.4*_: others	Nonexclusive	
	*D*_*16*_: collection events	*C*_*16.1*_: patient encounter OR *C*_*16.2*_: physical examination OR *C*_*16.3*_: diagnostics OR *C*_*16.4*_: treatment OR *C*_*16.5*_: progress note OR *C*_*16.6*_: communication OR *C*_*16.7*_: regulatory OR *C*_*16.8*_: others	Nonexclusive	
	*D*_*17*_: data linkage	*C*_*17.1*_: strategy OR *C*_*17.2*_: variable OR *C*_*17.3*_: completeness OR *C*_*17.4*_: cross-reference OR *C*_*17.5*_: none	Nonexclusive	
	*D*_*18*_: data preservation	*C*_*18.1*_: definite records XOR *C*_*18.2*_: indefinite records	Exclusive	
	*D*_*19*_: publish	*C*_*19.1*_: approval needed XOR *C*_*19.2*_: no approval needed	Exclusive	
	*D*_*20*_: informed consent	*C*_*20.1*_: not required XOR *C*_*20.2*_: general use XOR *C*_*20.3*_: all studies XOR *C*_*20.4*_: specific studies XOR *C*_*20.5*_: waiver XOR *C*_*20.6*_: others	Exclusive	

^a^FAIR: Findability, Accessibility, Interoperability, and Reusability.

^b^HMDC: health metadata catalog.

^c^DAC: Data Access Committee.

^d^BBMRI-MIABIS: Biobanking and Biomolecular Resources Research Infrastructure-Minimum Information About Biobank Data Sharing.

^e^CESSDA: Consortium of European Social Science Data Archives.

^f^DCAT: Data Catalog vocabulary.

^g^ECRIN-CRMDR: European Clinical Research Infrastructure Network – Clinical Research Metadata Repository.

^h^INSPIRE: Infrastructure for Spatial Information in Europe.

^i^PHIRI: Population Health Research Infrastructure.

^j^CDISC-SDTM: Clinical Data Interchange Standards Consortium - Study Data Tabulation Model.

^k^LOINC: Logical Observation Identifiers Names and Codes.

^l^OMOP: Observational Medical Outcomes Partnership.

^m^SNOMED: Systematized Nomenclature of Medicine.

^n^DICOM: Digital Imaging and Communications in Medicine.

^o^HL7 FHIR: Health Level 7 Fast Healthcare Interoperability Resources.

^p^IDMP: Identification for Medicinal Products.

^q^ISO800-110: International Organization for Standardization 800-110.

^r^CDM: common data model.

^s^ETL: extract, transform, load.

#### Data Findability

The meta-dimension prescribes that datasets orchestrated by an HMDC must be easily discoverable, requiring metadata assets to describe essential attributes of the decentral datasets [40]. The dimension *data source* (*D_1_*) refers to abstract categories for data classification in the catalog system. Following European Medicines Agency guidelines [43], and implementations in practice [49,50], patients’ health (*C_1.1_*) comprises datasets attributable to conditions. Examples are diseases, causes of death, prescriptions and dispensing of medicines, clinical measurements, genetic data, units of health care use, and all other similar patient-generated data, for example, wearables. Medical procedures (*C_1.2_*) encompass data describing hospital admission discharges, intensive care admissions, administration of vaccines or other injectables, medical operations, biomarker data, and diagnostic codes [43,44]. The latter includes, among others, the International Classification of Disease Code, the Major Comorbidity Code, and the Major Diagnostic Code. Medical products (*C_1.3_*) span categories like prescribed medicinal products for human use, contraception, indication for use, and medical device data. Others (*C_1.4_*) may refer to further data, for example, health care providers delivering diagnosis and treatment services [43]. The dimension *managerial details* (*D_2_*) captures crucial organizational metadata to be disclosed by the HMDC as part of a dataset’s metadata asset [24]. Above all, the data holder (*C_2.1_*) must be publicized, including contact details (ie, data steward). This entity sustains the record collection in an underlying dataset [60]. The origin (*C_2.2_*) of the data refers to the countries or geographical regions of their acquisition [60] and the language [44]. The characteristic of collection (*C_2.3_*) details the acquisition dates as well as all data assemblage information, except collection methodology and events. If the dataset has received a formal qualification (*C_2.4_*), this should also be disclosed in the metadata asset [43]. The same holds for sources of finance (*C_2.5_*) having sponsored the dataset creation [23,61], for example, data holder, public, industry, research, or patient organizations [44]. Naturally, other (*C_2.6_*) metadata attributes specifying managerial details are conceivable. The dimension *data type* (*D_3_*) describes broader content related categories applicable to the dataset. It mainly distinguishes datasets containing administrative (*C_3.1_* [62]), primary (*C_3.2_*) and secondary care (*C_3.3_* [63]), registry (*C_3.4_*), and other (*C_3.5_*) data types. Furthermore, *population information* (*D_4_*) as metadata attribute refers to the specifics of the records within a dataset [17]. The taxonomy narrows the dimension down to collected disease information (*C_4.1_*); particular population specifics (*C_4.2_*; eg, age groups); family linkages (*C_4.3_*; eg, household, parent-child, sibling, and not applicable) lifestyle factors (*C_4.4_*; eg, tobacco use, physical exercises, and diet); sociodemographic data (*C_4.5_*; eg, gender, ethnicity, education, and deprivation index); and (*C_4.6_*) catchment area coverage [17,43,44]. *Data sensitivity* (*D_5_*) addresses the identifiability of data subjects of whom records were collected. Records can be synthetic (*C_5.1_*), anonymized (*C_5.2_*), pseudonymized (*C_5.3_*), or contain personal data (*C_5.4_*) [15,41].

#### Data Accessibility

Once datasets are findable in the HMDC, their accessibility must be facilitated. By their design, HMDCs must ensure legal and ethical compliance of all data access and sharing processes in their health care ecosystems [19]. This particularly involves that datasets can be retrieved by authorized users only, implying rigor access control functions [5,41]. Accordingly, the dimension *catalogue accessibility* (*D_6_*) refers to access modalities for data consumers to use the HMDC. HMDCs can be public (*C_6.1_*) allowing anyone to browse metadata assets and discover datasets. Alternatively, HMDCs can be private (*C_6.2_*), limited to a certain number of users who have been formally authorized by a dedicated authority. In addition, hybrid (*C_6.3_*) forms exist. *Dataset accessibility* (*D_7_*) describes the access modalities for data consumers to published datasets [30]. The access can be free (*C_7.1_*), implying that data must at least be anonymized or synthetic to comply with legal and ethical guidelines [41,64]. Datasets can also require a formal request (*C_7.2_*) with a Data Access Committee (DAC) or a data steward deciding upon access requests [65,66]. HMDCs frequently require such requests of data consumers to be approved by their ethic committees before processing them within the ecosystem [66]. Moreover, data access can be strictly limited (*C_7.3_*) to a demarcated group of data consumers. Although, members of such limited groups also need to make formal requests for data access [66]. This implies that other (*C_7.4_*) data access modalities exist, especially combinations of *C_7.1_* to *C_7.3_*. *Access control* (*D_8_*) refers to the mechanisms implemented by HMDCs that facilitate the aforementioned decision-making by empowered entities [30]. The dimension specifies the entities who determine whether data consumers receive the requested datasets and are allowed to perform which kinds of processing activities [67]. It distinguishes the catalog operator (*C_8.1_*); internal DACs at the sides of the data holders (*C_8.2_* [65,66]); external DACs (*C_8.3_*) that are run centrally by an independent third party [23,66]; the absence of access control (*C_8.4_*; ie, free data [*C_7.1_*]); and any other forms (*C_8.5_*). Generally, access control in HMDC designs is crucial for maintaining data security, confidentiality, and compliance with legal and ethical constraints [67].

#### Data Interoperability

A core objective of HMDCs is to enable data consumers accumulating datasets across organizations, effectively, to create meaningful connections and analyses [7]. This makes data interoperability crucial [91]. To that end, HMDCs leverage specific standards described in this meta-dimension. Thereof, *programmatic discoverability* (*D_9_*) refers to the ability of data consumers to programmatically query, access, and retrieve metadata assets and search for their attributes. It is defined by the joint action Towards European Health Data Space (TEHDAS) [16] as the ability to identify, access, and understand health data by automated means. Associated approaches commonly involve application programming interfaces or similar programmatic methods to access and filter metadata in the HMDC [19]. Following the TEHDAS community [16], the dimension is narrowed down to the most frequent standards used by HMDCs. These are:

- Beacon (C_9.1_),
- Biobanking and biomolecular resources research infrastructure-minimum information about biobank data sharing (BBMRI-MIABIS; C_9.2_),
- Bio-image archive (C_9.3_),
- Consortium of European Social Science Data Archives (CESSDA; C9.4),
- Data catalog vocabulary (DCAT; C_9.5_),
- European Clinical Research Infrastructure Network – clinical research metadata repository (ECRIN-CRMDR; C_9.6_),
- FairSharing (C_9.7_),
- Infrastructure for Spatial Information in Europe (INSPIRE; C9.8),
- Population Health Research Infrastructure (PHIRI; C9.9), and
- Others (C_9.10_).

HMDCs typically adhere to one of those standards to ensure programmatic discoverability of published data offerings (ie, the metadata assets). The dimension *semantic interoperability* (*D_10_*) ensures that the precise format and meaning of datasets is preserved and understood, covering both semantic and syntactic aspects [68]. Similar to D_9_, the characteristics of this dimension encompass standards commonly applied by HMDCs. These are

- Clinical Data Interchange Standards Consortium - Study Data Tabulation Model (CDISC-SDTM; C10.),
- Logical Observation Identifiers Names and Codes (LOINC; C10.2),
- Observational Medical Outcomes Partnership (OMOP; C10.3),
- Orphanet (C10.4),
- Systematized Nomenclature of Medicine (SNOMED; C10.5 [16,68,69] and
- Others (C10.6).

Subsequently, the dimension *interoperable communication* (*D_11_*) comprises approaches implemented by HMDCs to facilitate seamless and effective data sharing between data holders and consumers [70]. Approaches typically used for interoperable communication are Digital Imaging and Communications in Medicine (DICOM; *C_11.1_*), Health Level 7 Fast Healthcare Interoperability Resources (HL7 FHIR; *C_11.2_*), Identification for Medicinal Products (IDMP; *C_11.3_*), and International Organization for Standardization (ISO) 800-110 (*C_11.4_*) [16,71]. As for the previous dimensions, other standards (*C_11.5_*) are conceivable. For *D_9_* to *D_11_*, detailed information is easily available in the web.

The following dimensions deal with “data harmonization*”* as a crucial aspect of data interoperability. They comprise HMDC design elements for standardizing disparate datasets. The purpose is to ensure consistency and coherence of all datasets classifiable to the same data type (*D_3_*). Data harmonization aims to create a unified and cohesive view on datasets, enhancing their allocation, sharing, and use [7]. The common data model (CDM; *D_12_*) describes the specifications relating to the structured representation of data records within datasets [5]. The CDM unfolds implications to the relationships between these records, as well as the rules and possibilities for data use. It defines how data consumers can access and process datasets, thus providing the foundation for data consistency, interoperability, and orchestration [10,72]. The taxonomy distinguishes the CDM type (*C_12.1_*) [10,43,72], the CDM references (*C_12.2_*), for example, websites or publications, and the release frequency of CDM specification updates (*C_12.3_*) [43]. Furthermore, information about datasets on their transformation status (extract, transform, load [ETL]) to a CDM should be provided [44]. This *ETL status* (*D_13_*) can be described as planned (*C_13.1_*), in progress (*C_13.2_*), or completed (*C_13.3_*), indicating the readiness of the dataset for use. Finally, HMDCs leverage *vocabularies* (*D_14_*) as sets of fixed terms, labels, or identifiers to describe and categorize the metadata assets [63]. Vocabularies facilitate understanding, discovery, and allocation of metadata with a consistently applied language [63,73]. The dimension distinguishes 10 characteristics for classifying vocabularies on the basis of pertinent literature: medicinal product (*C_14.1_*), cause of death (*C_14.2_*), quality of life measuring (*C_14.3_*), prescription (*C_14.4_*), dispensing (*C_14.5_*), indication (*C_14.6_*), procedures (*C_14.7_*), genetic data (*C_14.8_*), biomarker data (*C_14.9_*), and medical event (*C_14.10_*) [43,44,73].

#### Data Reusability

FAIR datasets must be created and documented in a way that allows reusage for different purposes. For HMDCs, this implies providing contextual information beyond the metadata dimensions associated with data findability [40]. *Collection methodology* (*D_15_*) encompasses characteristics that are associated with how data records were created [74]. Thereof, collection governance (*C_15.1_*) addresses information about data capture, demonstrating legal and ethical compliance [43]. This includes data quality checks and validation activities [75]. The latter may also refer to the question of whether the dataset allows access to the actual records. Furthermore, the collection process (*C_15.2_*) outlines how records in the dataset were created [74], for example, surveys, questionnaires, or data retrieval from hospital IS. Dataset updates (*C_15.3_*) disclose refreshment dates of datasets, for instance, fixed dates around the year [43]. Naturally, the collection methodology can contain other (*C_15.4_*) use case–specific characteristics as additional metadata attributes. Similar to *D_15_*, *collection events* (*D_16_*) narrows down the categories of incidents having triggered the creation of a record in the dataset [17]. The dimension comprises the characteristics of patient encounter (*C_16.1_*; eg, interactions with health care providers); physical examination (*C_16.2_*; eg, patient’s health examined by a professional); diagnostics (*C_16.3_*; eg, results of medical condition checks); treatment (*C_16.4_*; eg, documentation of conditions and treatment plans); progress notes (*C_16.5_*; eg, changes in patients’ health status, responses to treatment, or modifications of care plans); communication (*C_16.6_*; eg, information exchanged by health care providers); regulatory (*C_16.7_*; eg, legally required documentation of patients’ care); and others (*C_16.8_*) [43,44].

The dimension *data linkage* (*D_17_*) describes whether and how a dataset was created by linking others [43,44,76]. The metadata should disclose the linkage strategy (*C_17.1_*) which could be deterministic, probabilistic, or both. In addition, the used linkage variable (*C_17.2_*) should be published, along with the completeness of data linkage (*C_17.3_*). Ideally, the linked datasets should be cross-referred (*C_17.4_*) and, if applicable, their availability in the HMDC highlighted. In case no data linkage was applied, no corresponding metadata attribute is provided (*C_17.5_*). Furthermore, *data preservation* (*D_18_*) indicates whether records in the dataset are preserved indefinitely (*C_18.1_*) or, if not (*C_18.2_*), the time for which they are specified [77]. *Publishing constraints* (*D_19_*) provides information to data consumers whether an approval of the data holder (*C_19.1_*) is needed to publish results obtained from using the dataset or an approval is not needed (*C_19.2_*) [43]. In the former case, the kind of approval and the approval process should be described. Finally, metadata assets of HMDCs should reveal whether *informed consent* (*D_20_*) was obtained or needs to be obtained for data processing [78]. Generally, the characteristics not required (*C_20.1_*), required for general use (*C_20.2_*), required for all studies (*C_20.3_*), required for specific studies (*C_20.4_*), waiver (*C_20.5_*), and other (*C_20.6_*) are recommendable [43].

### Cocreated HMDC Use Cases

#### Overview

The usability, effectiveness, and accuracy of the taxonomy are amplified by 4 “illustrative scenarios” for HMDCs that demonstrate how the FAIR dimensions and characteristics are reflected in real-world use cases [39]. These use cases facilitate the taxonomy’s tangibility and the ascertainment of its practical implications, while triangulating the results. As such, they add depth and context to the taxonomy [39]. Originally, 6 abstract application scenarios were derived from recommendations of EMA [43]. Building upon this, we continuously developed and refined those scenarios on the basis of the insights gained during the taxonomy design iterations in general and the focus groups in particular. In the latter, we relied on the experts’ reflections on what they expect from an HMDC and whether our dimensions and characteristics meet their expectations, contradict them, or miss out on certain aspects. In the section about the deductive design iterations, we have already described how the focus groups were conducted. We ensured that the experts possess extensive expertise relevant to HMDC designs, either from a development (ie, technology or legal) or a user perspective (ie, data consumer or provider; Multimedia Appendix 3). Hence, the use cases followed a cocreation approach that was contextualized to the methodological process of the study [39]. Moreover, by being refined within multiple design iterations, the cocreated use cases have been triangulated and their relevance for HMDCs ensured. Following, their connection to the taxonomy is highlighted by direct references to dimensions (see *Taxonomy for FAIR HMDCs*). Additionally, I to VII refer to statements of experts who are listed in Multimedia Appendix 3. Reference citations of the literature and analysis objects demonstrate further exemplary sources having contributed to the final versions of the cocreated use cases.

#### Study Planning

Use case 1 is as follows: *a data consumer wants to identify suitable datasets for a planned study*.

The HMDC must enable data consumers to effectively identify datasets for medical research studies [15] by implementing the following process: First, a data consumer who wants to access the HMDC, needs to be authorized as a qualified user (*D_6_*; #I [50]). Second, this authorized user must be able to browse and filter published metadata assets to discover relevant datasets that fulfill specifications of an intended study (#V and VII [49,50]). For example, detailed data type [63] or population information of the metadata assets should be disclosed to enable verifying the relevance of datasets (*D_3_* and *D_4_*; #I, II, V, and VII [17,58]). Third, the HMDC must allow to check managerial details concerning information about the data holder, origin, collection, qualification, and financials (*D_2_* [43,50]), including the eligibility to receive synthetic, anonymized, pseudonymized, or personal data (*D_5_*; #IV [15]). Subsequently, the data consumer must be facilitated to perform a preliminary assessment of datasets regarding their relevance for the planned study (#III and VII). At this stage, a first list of candidates should be possible to be established. Ideally, the data consumer can access links (ie, cross-references) within the metadata assets to identify former studies which were performed with the same dataset, addressing similar research questions (*C_17.4_*; #II and VII [43,76]). Such studies are typically accessed outside the contextual boundaries of HMDCs (#IV). Finally, depending on the governance modalities of selected datasets (*D_7_*), the HMDC must enable the data consumer to request data access (#I-VII [30]). Therefore, an official data order needs to be submitted by the HMDC on behalf of the data consumer, containing specifications about the planned study and required documents, for example, protocols and ethical assessments (#I-III). With respect to the datasets accessibility constraints (*D_7_*) and their associated access control (*D_8_*) characteristics, the HMDC must forward data orders to the data holders (*C_8.2_* [66]), external third parties (*C_8.3_* [23]) or determine request permission or denial decisions itself (*C_8.1_* and *C_8.4_*; #I, III, and VI).

#### Study Assessment

Use case 2 is as follows: *a dataset is mentioned in a conducted study. The data consumer wants to evaluate this study based on the suitability of the datasets used therein.*

Given that the datasets used in a conducted study are available in the HMDC, data consumers must be enabled to verify, in retrospective, the suitability of these datasets (#I, II, and VII). To support such evaluations, the HMDC must provide different parts of the metadata asset, depending on the nature of the conducted study (#II and VII). For example, to assess the representativeness of the study population, the data consumer needs to examine qualitative metadata attributes (#II and VII), such as population information (*C_4.1_*-*C_4.5_* [17]); data type (*D_3_* [43,63]); collection methodology (*D_15_* [74]); and collection events (*D_16_* [17,43]). In addition, quantitative metadata such as the percentage of the population covered in the catchment area (*C_4.6_*) should be disclosed by the HMDC assets [17,43]. Furthermore, the data consumer might want to explore technical details to evaluate a study and its database, respectively (#I, II, and VII). Examples are the vocabularies used to define variables (*D_14_* [43,63]), the CDM according to which the used datasets are structured (*D_12_* [43]), the ETL status (*D_13_* [44]) and, if applied, any data linkage strategies (*D_17_* [76]). Moreover, cross-references should be listed in the metadata assets of the HMDC (*C_17.4_*) to allow identifying and obtaining lessons learned from other studies, where the same dataset was leveraged (#VII [76]). In doing so, the HMDC facilitates data consumers to identify strengths and limitations of datasets used in conducted studies.

#### Study Creation and Data Benchmarking

Use case 3 is as follows: *a data consumer writes a study protocol that requires to describe the underlying data sets and compare their characteristics.*

An HMDC must enable data consumers to easily access standardized metadata information about datasets that need to be specified and compared in a study protocol to be written (#I-VII). For HMDCs, this requires making attribute values of metadata assets directly and easily retrievable for data consumers to facilitate an efficient description and comparison of datasets (particularly, *D_1_*-*D_5_*; #VII). As such, when writing a study protocol, the data consumer can simply provide links to the metadata assets available in the HMDC, alongside with all kinds of other information that is interesting in the protocol’s context (#III and VII [43]). Providing such links is also beneficial because study readers could, in addition to basic metadata information (*D_1_*-*D_5_*), be interested in collection methodologies (*D_15_* [74]) and events (*D_16_* [17]), data preservation (*D_18_* [77]), consent requirements (*D_20_* [43,78]) as well as technical (*D_9_*-*D_14_*) and compliance specifics (*D_6_*-*D_8_*). Thus, HMDCs strengthen transparency and reproducibility of studies by facilitating an effective creation of study protocols (#III and V). At the same time, they support data benchmarking by providing detailed standardized metadata, potentially reaching beyond published datasets (#I-VII).

#### Data Analysis

Use case 4 is as follows: *a data consumer wants to benefit from the experience of others for the creation of a study’s programming script or statistical analysis.*

If a study relies on a CDM, the HMDC should enable the data consumer to identify, for published datasets, the ETL procedure (*D_13_*) from the dataset to the CDM (*D_12_*) [43]. Irrespective of whether data holders have converted their entire datasets, or only an extraction thereof, this information can support the development of the study script (#VII [43]). If the HMDC publishes cross-references of datasets (*C_17.4_*), the data consumer is also facilitated in finding further studies having investigated the same topic or used a comparable research design (#I, II, and VII [76]). These studies may disclose information on how to operationalize data variables, which offers the data consumer additional support in the development of a programming script (#I and VII). After data analysis, the HMDC may require the data consumer to record the developed script in a public repository and provide a link to the study protocol (see use case 3). This allows the HMDC to cross-reference it in the metadata assets of the datasets used (#V [43]). In doing so, transparency, reproducibility, and quality of studies are supported. Importantly, before publishing any study results, the HMDC should enable the data consumer to check whether approvals of data holders, of whom datasets were obtained, are required (*D_19_*; #IV and V [43]).

## Discussion

### Principal Findings

The taxonomy detailed in this work provides 20 dimensions and 101 characteristics to develop FAIR HMDCs, representing initial, yet actionable design knowledge to answer RQ1. Comprehensive description is achieved when amplifying the taxonomy in the light of the cocreated use cases that entail real-world requirements (RQ2). The generated design knowledge provides added value because HMDCs facilitate effective and efficient use of RWD for generating RWE. Thereby, the obtained results accentuate the integration of FAIR principles into HMDCs to ensure “findability, accessibility, interoperability, and reusability” of the RWD circulating within the underlying health care data ecosystem [40]. The design knowledge can be classified into scientific and managerial contributions, as outlined in the following sections.

### Interpretation of Findings

The taxonomy’s *scientific contributions* intensify previous work on data catalogs, paying particular attention to, first, their implementation as decentralized components within data ecosystems and, second, their application in health care peripheries. Consequently, some of the design elements conceptualized in this study draw from prior research about metadata catalogs, as shown in the section Research gap, objective, and questions. Concurrently, they further spin the red paths of HMDC developments with respect to European initiatives pushing health care data ecosystems forward, for example, IDERHA, Elixir, HealthData@EU, EUCAIM, TEHDAS, or EHDEN (Multimedia Appendix 1). Therefore, the taxonomy closes the identified research gap.

On the one hand, the conceptualized dimensions and characteristics of the taxonomy describe and classify attributes of “HMDC metadata assets.” First, they relate to attributes associated with data findability, such as data source, managerial details, the type of data records contained, data sensitivity, and population information. Second, they state crucial information pertaining to data reusability. This encompasses collection methodologies, data linkage, and preservation as well as consent and possible result publishing constraints. Third, the taxonomy emphasizes the need to specify data interoperability attributes in metadata assets. Among others, data standardization according to a CDM, the prescription of vocabularies, and the technological implementation of programmatic discoverability are important. On the other hand, the taxonomy contains design elements referring to more general data accessibility constraints pertaining to the “overall HMDC design.” They accentuate basic security and governance considerations regarding dataset and catalog accessibility as well as the access control framework. Conclusively, from a scientific viewpoint, the artifact provides fundamental design knowledge [31] that unfolds broad implications and a solid starting point for future research.

Regarding *managerial contributions*, the taxonomy enables health care practitioners (see Introduction section for target audience) to navigate more effectively in the largely unexplored field of HMDCs, particularly focusing on their application in health care data ecosystems across Europe. It helps both researchers and practitioners to anchor and communicate their work [41]. The taxonomy also represents a support tool for developing HMDCs, where the illustrative scenarios assume an accentuated role. They showcase how the design elements are reflected in real-world use cases [43]. In essence, these use cases amplify that the taxonomy supports common activities for planning, assessing, and conducting medical research studies as well as benchmarking and analyzing the underlying RWD.

Subsequently, the contributions of the study are discussed in the light of 3 major issues. These are (1) the exclusiveness of taxonomy characteristics; (2) the difference between HMDCs and centralized data catalogs; and (3) the absence of data quality and data-sharing incentives as explicit taxonomy dimensions.

Depending on the meta-dimension, the taxonomy contains nonexclusive characteristics, which might be accumulated, to facilitate the design of metadata assets, that is, data findability, interoperability, and reusability. Alternatively, the taxonomy has mutual exclusive characteristics to classify and distinguish HMDC designs with respect to their data security and governance approaches, that is, data accessibility. This mixture of exclusive and nonexclusive dimensions can foster the understanding of health care practitioners, while allowing for an easy alteration of the taxonomy [37]. Convertibility is vital because HMDCs represent a rapidly evolving and changing field, where new solutions vanish and emerge constantly.

Furthermore, even though the objective of the taxonomy is not to differentiate between centralized and decentralized data catalogs, as distinguished in the Theoretical Background section, it pinpoints their fundamental design commonalities and differences. The meta-dimensions concerning the design of the metadata assets are conceivable for both approaches in health care contexts (ie, findability, interoperability, and reusability). The reason is that, despite datasets being stored centrally within intraorganizational data catalogs [26], meaningful metadata need to be disclosed to their data users by means of the catalog offerings. Naturally, the same holds for decentralized catalogs. However, the taxonomy also shows design differences with respect to its meta-dimension data accessibility. While decentralized catalogs can have various combinations of characteristics in the associated dimensions, centralized health data catalogs exhibit one specific pattern of characteristics. They usually are exclusively private systems (*C_6.1_*) as their functionalities are only accessible to members of the operating organization. Similarly, dataset access is strictly limited to this specific group of predefined users (*C_7.3_*). Finally, access control lies solely with the organization operating the centralized catalog (*C_8.1_*).

As a last discussion point, the taxonomy contains neither dimensions associated with data quality nor incentives for data sharing. The reason is that these concepts, although important, are broad, multifaceted, and hardly explored, making a systematic categorization difficult. Generally, data quality involves subjective and context-dependent assessments [92], while incentives to share data are influenced by external, sociopolitical, and institutional factors [28]. Typically, HMDCs do not disclose any data quality metrics as those can barely be quantified and are subject to applied data types, formats, and standards [41]. Rather, HMDCs publish test samples consisting of synthetic or fully anonymized data that do not justify a dimension in the taxonomy. Similarly, incentivizing data sharing, for example, via price tags for datasets or mandatory citations, represents an unsolved problem [93]. To circumnavigate this issue, HMDCs commonly rely on membership fees and public funding. The former restricts data access to members of the HMDC operating organization. The latter compensates data providers through public funds, usually applied in preliminary stages. Naturally, other business models exist. However, both data quality indicators and data-sharing incentives represent underdeveloped fields requiring future research beyond the study’s design perspective [33]. Consequently, these concepts are not a part of the taxonomy, because they cannot be defined in universally applicable dimensions. Arguably, their inclusion would have overcomplicated the taxonomy and undermined its focus on actionable HMDC design knowledge.

### Limitations

The taxonomy is mainly subject to the following limitations. In the inductive iterations, results were derived from a potentially limited number of publications because of the emphasis on 4 main databases. Similarly, in the deductive iterations, the examined analysis objects might merely cover a snapshot of what was available at the time (ie, many analysis objects have been in progress), be outdated quickly, and not be conclusive. As for the SLR, the conclusiveness of analysis objects is particularly questionable because of, first, the focus on European ecosystem initiatives and, second, a possible negligence of many centralized health data catalogs. In the evaluative iterations, the experts might not have captured the full range of relevant perspectives on HMDCs and are limited in number. Furthermore, the research design comprises certain limitations per se. As it is with qualitative research, taxonomy building requires substantial generalizations and simplifications of intricate and interdisciplinary content [83]. Although countermeasures were taken (see Methods section), these factors imply interpretative biases inevitably incorporated into the results [41], for example, extracting design elements from public data. Moreover, as shown in the Theoretical Background section, new HMDCs must be expected to arise constantly, while others are likely to disappear with a high frequency. Hence, the taxonomy must be altered swiftly. To conclude, the taxonomy provides first actionable design knowledge about HMDCs but requires continuous triangulation of design elements by future research.

### Conclusions

Despite the limitations, the scientific and managerial contributions of this study unfold broad implications, which are formulated as recommendations for future research. Generally, HMDCs should be increasingly investigated in practice, for example, by more in-depth case studies. On the one hand, it is of utmost importance to keep track of the rapidly evolving HMDC-related initiatives in Europe. Their conceptual and technical advancements should be analyzed and evaluated constantly against the background of the taxonomy design elements, deriving the need for modifying dimensions and characteristics. On the other hand, by incorporating worldwide efforts toward health care data ecosystems and HMDCs, the scope of the taxonomy can be expanded and design knowledge beyond European jurisdictions can be created. In this regard, it is important to mention that the generated design knowledge about European HMDCs already entails such global implications. The FAIR dimensions of the taxonomy state fundamental characteristics of health data FAIR, making it universally relevant. In other words, the taxonomy conveys generally conceivable options for using catalog functionalities and underlying metadata assets. In addition, it outlines how to design those health metadata assets meaningfully. Therefore, despite the European focus, the taxonomy addresses global challenges with regards to health data sharing and metadata catalog designs, underlining its broad implications. Nevertheless, further research is essential, because HMDCs represent the fulcrum for allocating, exchanging, and using RWD to effectively generate RWE in emerging health care data ecosystems.

## Appendix

Multimedia Appendix 1 Leading European initiatives toward health care data ecosystems.

Multimedia Appendix 2 Intermediary results of the iterative research process and key references.

Multimedia Appendix 3 List of focus group members and distribution to sessions.

## Abbreviations

CDM: common data model
DAC: Data Access Committee
E2C: empirical-to-conceptual
EHDEN: European Health Data and Evidence Network
EHDS: European Health Data Space
EHDS2: European Health Data Space 2
ETL: extract, transform, load
EU: European Union
EUCAIM: European Federation for Cancer Images
FAIR: findability, accessibility, interoperability, and reusability
HMDC: health metadata catalog
IDERHA: Integration of Heterogeneous Data and Evidence towards Regulatory and Health Technology Assessment Acceptance
IS: information systems
PRISMA: Preferred Reporting Items for Systematic Reviews and Meta-Analyses
RQ: research question
RWD: real-world data
RWE: real-world evidence
SLR: structured literature review
TEHDAS: Towards European Health Data Space

## Data Availability

Data sharing is not applicable to this paper as no datasets were generated or analyzed during this study.
