# Supplementary material for: Exploring Metadata Catalogs in Health Care Data Ecosystems: Taxonomy Development Study
Source: JMIR Form Res. 2025 Feb 18;9:e63396. doi: 10.2196/63396 (PMC11888085; doi:10.2196/63396)
Supplement: Multimedia Appendix 1 [file formative_v9i1e63396_app1.docx]

**Multimedia Appendix 1: Leading European initiatives towards healthcare data ecosystems**

| **Initiative** | **EHDEN** | **HealthData@EU (EHDS2)** | **EUCAIM** | **ELIXIR** | **IDERHA** |
| --- | --- | --- | --- | --- | --- |
| Start Date | 01.11.2018 | 01.10.2022 | 01.01.2023 | 01.11.2007 | 01.04.2023 |
| Duration | 72M | 24M | 48M | 2007 (November) onwards | 60M |
| Sponsor | EU and EFPIA | EU | EU + | EU, IMI | Innovative Health Initiatives |
| Leading organizations | Janssen Pharmaceutica NV &  Erasmus Univ. Med. Centre | Health Data Hub | European Institute for Biomedical Imaging Research (EIBIR) | European Molecular Biology Laboratory | Fraunhofer ITMP and Johnson & Johnson Medical GmbH |
| Partners | 25 | 17 | 76 | 36 | 33 |
| Use Cases | Study: Transforming Estonian health data to the Observational Medical Outcomes Partnership (OMOP) Common Data Model within a data ecosystem approach. | Infectious disease surveillance (AMR): Demonstrate the feasibility of using the EHDS to carry out infectious disease surveillance, focusing on antimicrobial resistance. | Developing a public catalogue for cancer imaging datasets based on the repositories of the EU-funded AI for Health Imaging projects. The catalogue applies a common metadata schema. | Enable cross-border data analysis with a broad array of partners from EU life science industry by embedding common practices across the whole European Research Area via the ELIXIR healthcare data ecosystem. | Leverage AI/ML approaches within a healthcare data ecosystem for personalized early risk profiling using multimodal data. |
|  | NETWORK STUDY: Supporting Pharmacovigilance Signal Validation and Prioritization with Analyses of Routinely Collected Health Data. | Thrombosis for COVID-19 patients: Foster a better understanding of the risks of thrombosis for COVID-19 patients. | Build a federated searching tool to understand the information available at the federated data providers. |  | Leverage AI/ML approaches within a healthcare data ecosystem for personalized malignancy risk prediction using CT scan image data. |
|  | Study: Background Rates of AESI for Vaccines. | Colorectal cancer: Mobilise and chain clinical and genomic data to enhance the understanding of colorectal cancer. | Full integration with the Life Science Login Authentication and Authorization Infrastructure. | Create a toolkit for robust, reproducible, and green software and workflows. | Create AI/ML personalized diagnosis of existing malignancy using CT scan image data. |
|  | Health Technology Assessment (HTA) Use Case: Chronic Obstructive Pulmonary Disease (COPD). | Covid-19 testing, vaccination and hospitalisation: Compare COVID-19 testing, vaccination and hospitalisation between the general population and vulnerable subpopulations. | Reusing and adding value to key components of EU-funded research projects and infrastructure in the field of cancer. | Establish network of partners in Europe and internationally to drive global competitiveness and sustainability of the emerging healthcare data ecosystem. | Establish remote late-stage lung cancer patient monitoring using wearables and digital engagement at home. |
| Context | Health data standardization, sharing and utilization in general | Health data sharing and utilization in general | Health data sharing in oncology imaging | Health data sharing and utilization in life sciences | Health data sharing and utilization for lung cancer studies |
| Project description | EHDEN implements a large-scale healthcare data ecosystem within Europe by harmonizing, standardizing, and sharing health data from multiple sources between participants, using OMOP-CDM. To overall objective is to improve research outcomes. | Health Data @ EU builds a pilot version of the European Health Data Space 2 (EHDS2) infrastructure for the **secondary use** of health data which aims to serve medical research, health innovation, policy making and regulatory purposes. | EUCAIM is developing a pan-EU healthcare data ecosystem for cancer image AI research and clinical practices. | ELIXIR establishes a governing ecosystem that enables researchers to access, share, and analyse life science data. It also aims to improve the value and impact of life science research on public health, the environment, and the European economy. | IDERHA develops a European healthcare data ecosystem in close alignment with the EHDS. It aims to enable researchers sharing and utilizing lung cancer data for secondary use, while also facilitating regulatory approvals and Health Technology Assessments (HTA). |
| Link | [www.ehden.eu/](http://www.ehden.eu/) | <https://ehds2pilot.eu/> | www.cancerimage.eu/ | <https://elixir-europe.org/> | [www.iderha.org/](http://www.iderha.org/) |

Note: Further important initiatives with regards to HMDC in (European) healthcare ecosystems are listed in Table 3 of the paper.
