# Supplementary material for: Exploring Metadata Catalogs in Health Care Data Ecosystems: Taxonomy Development Study
Source: JMIR Form Res. 2025 Feb 18;9:e63396. doi: 10.2196/63396 (PMC11888085; doi:10.2196/63396)
Supplement: Multimedia Appendix 2 [file formative_v9i1e63396_app2.docx]

**Multimedia Appendix 2: Intermediary results of the iterative research process**

**TII: Taxonomy after Inductive Iterations (C2E1, C2E2)**

The morphologic box of TII contains the version of dimensions and characteristics after the inductive iterations (i.e., analysing related work (C2E1) and conducting the structured literature review (C2E2)).

|  | Dim. | Characteristics | e/n |
| --- | --- | --- | --- |
| Data Findability | data search | full text / semantic / fuzzy / natural language / filters / multiple | e |
|  | data source | patients / procedures / products / others | n |
|  | data type | primary care / secondary care / registries / other | e |
|  | metadata management | schema / semi-structured / customized / inference / other | e |
|  | population information | disease / human linkages / lifestyle factors / societal factors / catchment area | n |
| Data Accessibility | catalogue access | public / hybrid / private | e |
|  | data governance | policy enforcement / data quality metrics / compliance tracking / access control / auditing and logging | n |
|  | dataset access | free / formal request / strictly limited / others | e |
|  | data access control | role-based / attribute-based / policy-based / other | e |
|  | data security | pseudonymization / anonymization / both / none | e |
|  | data output | unprocessed data / aggregated data / standardized data / multiple | e |
| Data Interoperability | integration | API / ETL tools / connectors / middleware / multiple / others | e |
|  | program. discoverability | Beacon / BBMRI-MIABIS / Bio-image / CESSDA / DCAT / ECRIN-CRMDR / FairShairing / INSPIRE / PHIRI / others | n |
|  | semantic interoperability | CDISC-SDTM / LOINC / OMOP / Orphanet standards / SNOMED / others | n |
|  | interop. communication | DICOM / HL7 FHIR / IDMP / ISO 800-110 / others | n |
|  | data model | entity / data type / relationships / attributes / standard / other | n |
|  | vocabularies | medicinal product / cause of death / quality of life measurement / prescription / dispensing / indication / procedures / genetic data / biomarker data / medical event | n |
| Data Reusability | data users | academic / industry / governments / others | n |
|  | data collection | patient encounter / physical examination / diagnostics / treatment / progress note / communication / regulatory / others | n |
|  | data linkage | strategy / variable / completeness / cross-reference / none | n |
|  | preservation | definite records / indefinite records | e |
|  | consent | specific / broad / dynamic / meta / none / others | e |

**TDI: Taxonomy after Deductive Iteration (E2C1)**

The morphologic box of TDI contains the version of dimensions and characteristics after the first deductive iteration (i.e., general analysis of representatives from practice (E2C1)).

|  | Dim. | Characteristics | | | e/n |
| --- | --- | --- | --- | --- | --- |
| Findability | data discovery | search / navigation / recommendation / filter / multiple | | | e |
|  | data source | patients / procedures / products / others | | | n |
|  | data type | admin / primary care / secondary care / registries | | | e |
|  | metadata management | schema / semi-structured / customized / inference / other | | | e |
|  | population information | disease / family linkages / lifestyle factors / population / sociodemographic / catchment area coverage | | | n |
| Access | catalogue access | public / hybrid / private | | | e |
|  | data governance | policy enforcement / data quality metrics / compliance tracking / access control / auditing and logging | | | n |
|  | dataset access | free / formal request / strictly limited / others | | | e |
|  | user interface | web-based / API-driven / mobile-responsive / multiple / other | | | e |
|  | data access control | role-based / attribute-based / policy-based / other | | | e |
|  | data sensitivity | pseudonymization / anonymization / both / none | | | e |
| Interoperability | integration | API / ETL tools / connectors / middleware / multiple / others | | | e |
|  | program. discoverability | Beacon / BBMRI-MIABIS / Bio-image / CESSDA / DCAT / ECRIN-CRMDR / FairShairing / INSPIRE / PHIRI / others | | | n |
|  | semantic interoperability | CDISC-SDTM / LOINC / OMOP / Orphanet standards / SNOMED / others | | | n |
|  | interop. communication | DICOM / HL7 FHIR / IDMP / ISO 800-110 / others | | | n |
|  | CDM | type / reference / release frequency | | | n |
|  | ETL status | planned / in progress / completed | | | e |
|  | vocabularies | medicinal product / cause of death / quality of life measurement / prescription / dispensing / indication / procedures / genetic data / biomarker data / medical event | | | n |
| Reusability | collection methodology | governance / process / dataset updates / others | | | n |
|  | collection events | patient encounter / physical examination / diagnostics / treatment / progress note / communication / regulatory / others | | | n |
|  | data linkage | strategy / variable / completeness / cross-reference / none | | | n |
|  | data pres. | definite records / indefinite records | | | e |
|  | informed consent | | not required / general use / all studies / specific studies / waiver / others | e | |

**TEDI: Taxonomy after Evaluative and Deductive Iterations (Eval1, E2C2)**

The morphologic box of TEDI contains the version of dimensions and characteristics after the first evaluative and the second deductive iterations (i.e., focus group session one (Eval1) and specific analysis of representatives from practice based on Eval1 (E2C2)).

|  | Dim. (D_n_) | Characteristics (C_nm_) | e/n |
| --- | --- | --- | --- |
| Findability | (*D_1_*) data source | patients / procedures / products / others | n |
|  | (*D_2_*) mngt. details | holder / origin / collection / qualification / financials / others | n |
|  | (*D_3_*) data type | admin / primary care / secondary care / registries | e |
|  | (*D_4_*) population information | disease / family linkages / lifestyle factors / population / sociodemographic / catchment area coverage | n |
|  | (*D_5_*) sensitivity | raw / anonymized / pseudonymized / personal | e |
| Accessibility | (*D_6_*) catalogue access | public / hybrid / private | e |
|  | (*D_7_*) data access | free / formal request / strictly limited / others | e |
|  | (*D_8_*) formal request process | catalog operator / internal DAC / external DAC / none / others | n |
| Interoperability | (*D_9_*) program. discoverability | Beacon / BBMRI-MIABIS / Bio-image / CESSDA / DCAT / ECRIN-CRMDR / FairShairing / INSPIRE / PHIRI / others | n |
|  | (*D_10_*) semantic interoperability | CDISC-SDTM / LOINC / OMOP / Orphanet standards / SNOMED / others | n |
|  | (*D_11_*) interop. communication | DICOM / HL7 FHIR / IDMP / ISO 800-110 / others | n |
|  | (*D_12_*) CDM | type / reference / release frequency | n |
|  | (*D_13_*) ETL status | planned / in progress / completed | e |
|  | (*D_14_*) vocabularies | medicinal product / cause of death / quality of life measurement / prescription / dispensing / indication / procedures / genetic data / biomarker data / medical event | n |
| Reusability | (*D_15_*) collection methodology | governance / process / dataset updates / others | n |
|  | (*D_16_*) collection events | patient encounter / physical examination / diagnostics / treatment / progress note / communication / regulatory / others | n |
|  | (*D_17_*) data linkage | strategy / variable / completeness / cross-reference / none | n |
|  | (*D_18_*) data pres. | definite records / indefinite records | e |
|  | (*D_19_*) publish | approval needed / no approval needed | e |
|  | (*D_20_*) informed consent | not required / general use / all studies / specific studies / waiver / others | e |

The TEDI version is very similar to the final taxonomy presented in the main paper, except for a few wording issues and the characteristic “other” in the dimension data type (*D_3_*). See Sections 2 and 3 of the manuscript for more details.

**Key references per dimension of final taxonomy (Eval2)**

The following tables show the key references (i.e., literature and analysis objects) that contributed to drafting the *final* versions of the taxonomy’s dimensions and characteristics. Importantly, not every identified analysis object (and literature source) was useful in conceptualizing a dimension with its characteristics. Hence, for each dimension, a subset of sources unfolded relevance only. Consequently, the dimensions and characteristics of the taxonomy represent empirically grounded best practices for HMDC that were conceptualized and evaluated in our study, leveraging the combination of research methods described in Section 2 of the paper. Section 3 of the paper presents the ultimate versions.

|  | **Dim. (D_n_)** | **Key References** | |
| --- | --- | --- | --- |
|  |  | **Key Literature Sources** | **Key Analysis Objects** |
| Findability | (*D_1_*) data source | - European Medicines Agency (2022a) [43] - European Medicines Agency (2022b) [44] - Oliveira et al. (2019) [28] | - BBMRI-ERIC Data Directory [45] - Catalogue of Mental Health Measures [46] - Compendium Data Catalog for Healthcare [47] - EMIF Data Catalogue [49] - Lifebit Precision Medicine Data Catalogue [57] - Maelstrom Research Data Catalogue [58] |
|  | (*D_2_*) management details  *Former: metadata management (see TII)* | - European Medicines Agency (2022a) [43] - European Medicines Agency (2022b) [44] - Labadie et al. (2020) [24] - McCoy et al. (2009) [61] - Oliveira et al. (2019) [28] - Shi et al. (2017) [60] | - All analysis objects listed in Table 4 of the paper contain some form of (meta-) data-related management details as per the characteristics defined in the taxonomy. |
|  | (*D_3_*) data type | - European Medicines Agency (2022a) [43] - Pereira et al. (2023) [63] - Yurkovich et al. (2015) [62] | - All analysis objects listed in Table 4 of the paper contain some form of data type characteristics as defined in the taxonomy. - However, this information was not accessible for all catalogues. |
|  | (*D_4_*) population information | - Bergeron et al. (2018) [17] - European Medicines Agency (2022a) [43] - European Medicines Agency (2022b) [44] | - BBMRI-ERIC Data Directory [45] - European Health Information Portal [51] - HealthRI Data Catalogues [53] - IQVIA Health Data Catalogue [55] - Maelstrom Research Data Catalogue [58] - YODA Trials Data Catalogue [59] |
|  | (*D_5_*) data sensitivity  *Former: data security (see TII)* | - Scheider et al. [41] - Oliveira et al. (2019) [28] | - All analysis objects listed in Table 4 of the paper contain some form of data sensitivity characteristics as defined in the taxonomy. |

|  | **Dim. (D_n_)** | **Key References** | |
| --- | --- | --- | --- |
|  |  | **Key Literature Sources** | **Key Analysis Objects** |
| Accessibility | (*D_6_*) catalogue accessibility  *Former: cat. access (see TII)* | - Scheider et al. [41] - Swertz et al. (2022) [5] | - All analysis objects listed in Table 4 of the paper contain some form of catalogue accessibility characteristics as defined in the taxonomy. |
|  | (*D_7_*) dataset accessibility | - Almeida and Oliveira (2024) [30] - Alvarellos et al. (2023) [42] - Brunswick (2019) [64] - Swertz et al. (2022) [5] - Yang et al. (2013) [65] | - EHDEN Portal [48] - EUCAIM Cancer Image Europe [50] - Fjelltopp Data Catalogues for Health [52] - Helsedata Explore Data Sources [54] - IDERHA^a^ - Kraken Health Pilot [56] |
|  | (*D_8_*) access control  *Former: data access control and data governance (see TII)* | - Almeida and Oliveira (2024) [30] - Alvarellos et al. (2023) [42] - Scheider et al. [14] - Munoz-Arcentales et al. (2019) [67] - Oliveira et al. (2019) [28] - Shabani and Borry (2016) [66] - Yang et al. (2013) [65] | - All analysis objects listed in Table 4 of the paper contain some form of access control characteristics as defined in the taxonomy. |

|  | **Dim. (D_n_)** | | **Key References** | | |  |
| --- | --- | --- | --- | --- | --- | --- |
|  |  |  | **Key Literature Sources** | **Key Analysis Objects** | |  |
| Interoperability | (*D_9_*) program. discoverability | | - Almeida et al. (2023) [19] - TEHDAS (2022) [16] | - BBMRI-ERIC Data Directory [45] - Fjelltopp Data Catalogues for Health [52] | |  |
|  | (*D_10_*) semantic interoperability | | - de Mello et al. (2022) [69] - Ngouongo et al. (2013) [68] - TEHDAS (2022) [16] | - IDERHA^a^ | |  |
|  | (*D_11_*) interop. communication | | - Iroju et al. (2013) [70] - Roehrs et al. (2018) [71] - TEHDAS (2022) [16] | - No concrete information retrievable. | |  |
|  | (*D_12_*) CDM  *Former: data model (see TII)* | | - European Medicines Agency (2022a) [43] - European Medicines Agency (2022b) [44] - Kent et al. (2021) [72] - Lovestone (2020) [10] - Swertz et al. (2022) [5] | - EUCAIM Cancer Image Europe [50] - IDERHA^a^ | |  |
|  | (*D_13_*) ETL status | | - European Medicines Agency (2022a) [43] - European Medicines Agency (2022b) [44] | - No concrete information retrievable. | |  |
|  | (*D_14_*) vocabularies | | - European Medicines Agency (2022a) [43] - European Medicines Agency (2022b) [44] - Ivanovic and Budimac (2014) [73] | - No concrete information retrievable. | |  |
|  | | **Dim. (D_n_)** | | **Key References** | | |
|  |  |  |  | **Key Literature Sources** | **Key Analysis Objects** | |
| Reusability | | | (*D_15_*) collection methodology  *Former: data collect. (see TII)* | - European Medicines Agency (2022a) [43] - Hentschel (1999) [74] - Tijhuis et al. (2019) [75] | - BBMRI-ERIC Data Directory [45] - Compendium Data Catalog for Healthcare [47] - EUCAIM Cancer Image Europe [50] |  |
|  |  |  | (*D_16_*) collection events  *Former: data collect. (see TII)* | - Bergeron et al. (2018) [17] - European Medicines Agency (2022b) [44] | - Compendium Data Catalog for Healthcare [47] - European Health Information Portal [51] - Maelstrom Research Data Catalogue [58] |  |
|  |  |  | (*D_17_*) data linkage | - Bohensky et al. (2010) [76] - European Medicines Agency (2022a) [43] - European Medicines Agency (2022b) [44] | - IQVIA Health Data Catalogue [55] |  |
|  |  |  | (*D_18_*) data pres.  *Former: data pres. (see TII)* | - Rasmussen and Blank (2007) [77] | - No concrete information retrievable. |  |
|  |  |  | (*D_19_*) publish | - European Medicines Agency (2022a) [43] | - No concrete information retrievable. |  |
|  |  |  | (*D_20_*) informed consent  *Former: consent (see TII)* | - Angrist (2009) [78] - Scheider et al. [41] - European Medicines Agency (2022a) [43] | - Kraken Health Data Pilot [56] |  |

^a^ The metadata catalogue is not publicly accessible yet.
