# Supplementary material for: Exploring Metadata Catalogs in Health Care Data Ecosystems: Taxonomy Development Study
Source: JMIR Form Res. 2025 Feb 18;9:e63396. doi: 10.2196/63396 (PMC11888085; doi:10.2196/63396)
Supplement: Multimedia Appendix 3 [file formative_v9i1e63396_app3.docx]

**Multimedia Appendix 3: List of focus group members and distribution to sessions**

The following table lists the focus group members who participated in the evaluation of the taxonomy and the co-creation of use cases during the 4^th^ and 6^th^ iterations. It shows the EU initiatives the experts were recruited from (see Multimedia Appendix 1) and whether these initiatives contain an operative HMDC or one that is still in progress.

| Exp. | Professional background | Roles in EU Initiatives | general | | | | political | |
| --- | --- | --- | --- | --- | --- | --- | --- | --- |
|  |  |  | EHDEN^a^ | Elixir^a,b^ | EUCAIM^a^ | IDERHA^c^ | HD@EU^d^ | TEHDAS^d^ |
| I | Software Engineering | - Software Developer - Solution Architect |  | X |  | X |  |  |
| II | Computer Science | - Product Owner - Solution Architect | X |  |  |  |  |  |
| III | Computer Science, Data Governance | - Data Compliance Officer - Solution Architect |  |  | X |  |  |  |
| IV | Health Informatics | - Expert in health data standards and interoperability |  |  |  |  | X | X |
| V | Software Engineering | - Solution Architect - Data Protection Officer |  |  |  | X |  |  |
| VI | Healthcare Provisioning | - Medical Expert - Data Steward | X |  |  | X |  |  |
| VII | Healthcare Research | - Data Scientist |  |  |  | X |  |  |

^a^ operative

^b^ Elixir supports the data catalogs called “BioSamples” and “FAIRsharing” (see Table 4 of main paper). They aim at standardizing the documentation and accessibility of biological data and samples.

^c^ in-progress

^d^ political initiative (no technical artifact)

The table below shows the distribution of experts to focus group sessions per iteration.

| Design iteration | Experts per session | | | No. of experts in focus group |
| --- | --- | --- | --- | --- |
|  | Session 1 | Session 2 | Session 3 |  |
| 4^th^ | 3 | 2 | - | 5 |
| 6^th^ | 4 | 1 | 2 | 7 |
